# Editorial: *Candida* spp.-transmission, pathogenesis, host-pathogen interaction, prevention, and treatment

**DOI:** 10.3389/fmicb.2023.1258837

**Published:** 2023-08-02

**Authors:** Sameh S. M. Soliman

**Affiliations:** College of Pharmacy, University of Sharjah, Sharjah, United Arab Emirates

**Keywords:** *Candida*, antifungal resistance, fungal infection, prevalence, mutation, aneuploidy

*Candida* spp. are a group of yeast-like fungi that are commonly found in the environment and on the skin and mucous membranes of humans (Talapko et al., 2021). While they generally do not cause harm under normal conditions, they can become opportunistic pathogens in certain situations such as in the case of immunocompromised patients. *Candida* spp. are transmitted through direct contact with infected individuals or contaminated surfaces. Common modes of transmission include person-to-person contact, sexual contact, and contact with contaminated objects or surfaces. In healthcare settings, *Candida* spp. can also spread through contaminated medical equipment and devices (Silva et al., 2012). The ability of *Candida* spp. to cause disease depends on several factors such as the virulence of specific *Candida* spp., the immune status of the host, and the local environment (Mba and Nweze, 2020). *Candida* spp. have developed various mechanisms to evade the host's immune defenses and establish infection. They can produce virulent factors such as adhesins, which allow them to adhere to host cells and surfaces. *Candida* can also form biofilms, making them more resistant to the host's immune system and antifungal treatments. Preventing *Candida* infections may involve several strategies including maintaining good hygiene, avoiding unnecessary antibiotic use, controlling underlying medical conditions, and infection control in healthcare settings (Ture and Alp, 2018). There are continuous efforts in understanding the invasion, pathogenesis, and resistance mechanisms by these fungal spp., hence driving toward effective prevention and treatment. In this Research Topic, we aim to explore the scientific gaps associated with *Candida* spp. including mode of transmission, pathogenesis and resistance mechanisms, host-pathogen interaction and immune evasion mechanisms, and prevention and possible treatment strategies against these fungal infections.

The first article of this Topic by Jain et al. carried out molecular dissection of *TAC1* gene, which encodes the transcriptional activator of ABC transporters Cdr1p and Cdr2p in *Candida albicans*. Away of the N-terminal DNA Binding Domain (DBD) of Tac1p that interacts with the Drug Responsive Element (DRE) present in the upstream promoter region of *CDR1* and *CDR2* genes and the C-terminal Acidic Activation Domain (AAD) that interacts with the TATA box Binding Protein (TBP), by homology modeling, the authors identified a Middle Homology Region (MHR) of Tac1p that acts as a probable xenobiotic binding domain (XBD), and propose its importance in drug resistance. MHR interaction with small molecules may lead to a change in conformation in the overall structural assembly of Tac1p on CDR promoter resulting in differential gene expression. In this regards, strains CAF 4–2 (containing wild type *TAC1*), DSY2906 (*TAC1* deletion), DSY2925 (wild type *TAC1* revertant), and DSY2926 (GOF *TAC1* revertant) were tested with Fluphenazine, a xenobiotic compound that causes transient up-regulation of *CDR* gene expression (Cortez et al., 2008). Only strain DSY2906 showed no change in the mRNA expression levels of *CDR1* and *CDR2* genes, suggesting that Tac1p might act as a xenobiotic receptor. Furthermore, a significant change in the expression of lipid profiling genes such as *ERG3, ERG6, ERG11* and stress response genes such as *PDE2, SOD1, SOD5, SSK-1, TTR1*, and *UPC2*, genes contain DRE sequences, were observed in Fluphenazine-induced *C. albicans* strains.

Li et al. study aimed to develop and validate a serological diagnosis system using novel clinical biomarkers to predict the existence of invasive candidiasis (IC) in immunocompetent intensive care unit (ICU) patients using multivariate regression analysis. A total of 1,841 ICU patients were employed in this study. The authors showed that C-reactive protein-to-albumin ratio (CAR) and neutrophil-to-lymphocyte ratio (NLR) were higher, while the prognostic nutritional index (PNI) was lower in IC group than in non-IC group. Patients in the IC group showed higher rates of diabetes mellitus (DM), sepsis, and solid tumor than those in the non-IC group (*p* < 0.05). Furthermore, patients with IC had a longer hospital stay (14.5 vs. 9 days, *p* < 0.001) and ICU stay (10 vs. 7 days, *p* < 0.001) than those without IC. CAR, and NLR were positively correlated (*p* < 0.001), while PNI was inversely correlated (*p* < 0.001) with Sequential Organ Failure Assessment (SOFA) scores.

In Fan et al. the authors identified the high prevalence of fluconazole resistant *Candida tropicalis* among candiduria samples in China. A 12-year (2010–2021) laboratory-based surveillance and fluconazole susceptibility study of *C. tropicalis* was conducted. The nucleotide mutations in *ERG11* gene were also compared between resistant- and wild-type strains. The study revealed a high azole resistant rate and earlier detection in candiduria strains than those causing IC. A total of 519 *C. tropicalis* strains were isolated; of them, 69.9% were isolated from urine samples and 30.1% from invasive infections, in addition to 16.5% isolates were fluconazole resistant. They also reported that *ERG11* sequencing from fluconazole-resistant strains revealed the prevalence of A395T/W mutations. Isolates carrying Y132F substitution were highly resistant to fluconazole with MIC_50_ ~256 μg/ml.

Yang et al. conducted a retrospective observational study on the clinical features and treatment plan of hematological disease patients associated with *Candida tropicalis* bloodstream infections. A total of 26 patients were enrolled, and univariate analysis using Kaplan–Meier analysis and multivariate analysis using Cox regression model were conducted. The authors showed that hematological disease patients with *C. tropicalis* bloodstream infections exhibited a high mortality rate, while the early antifungal therapy significantly reduced the mortality rate. All patients had neutropenia, while 88.5% had hematologic malignancies. Seven, 15, and 8 patients were treated separately with azole, echinocandin, or amphotericin B, respectively; of them 5, 3, and 2 failed treatment and died, respectively. Univariate analysis showed that septic shock, Pitt bacteremia, procalcitonin, plasma (1,3)- β-D glucan level, serum albumin level, and time from fever/neutropenia to treatment were the major risk factors associated with deaths, while multivariate analysis showed that septic shock (*p* = 0.006) was an independent risk factor. Furthermore, antifungal susceptibility was determined from patient blood cultures. All isolates were sensitive to flucytosine and amphotericin B, while 41.7, 50, and 41.7%, were resistant to fluconazole, itraconazole and voriconazole, respectively. However, we believe that the study is limited by the number of included patients in the study.

Sun et al. suggested that chromosome aneuploidy provides a rapid and reversible mechanism of drug tolerance and cross-tolerance in *Candida parapsilosis*. They observed that chromosome 5 trisomy was the major mechanism of tolerance to caspofungin and cross-tolerance to 5-flucytosine, while point mutations in the target *FKS* genes were rare. This tolerance is due to an increase in the copy number and expression of *CHS7* gene, which encodes chitin synthase. The authors also suggested that the inherent instability of aneuploidy caused an unstable drug tolerance.

We hope that the readers will benefit from this Research Topic in the design of a future investigation related to pathogenesis, resistance and diagnosis or clinical intervention in common *Candida* spp. or newly emerging spp.

## Author contributions

SS: Conceptualization, Data curation, Formal analysis, Funding acquisition, Investigation, Methodology, Project administration, Resources, Supervision, Validation, Visualization, Writing—original draft, Writing—review and editing.
